# Sequencing of Side-Chain Liquid Crystalline Copolymers by Matrix-Assisted Laser Desorption/Ionization Tandem Mass Spectrometry

**DOI:** 10.3390/polym11071118

**Published:** 2019-07-01

**Authors:** Savannah R. Snyder, Wei Wei, Huiming Xiong, Chrys Wesdemiotis

**Affiliations:** 1 Department of Chemistry, The University of Akron, Akron, OH 44325, USA; 2 Department of Polymer Science, School of Chemistry and Chemical Engineering, Shanghai Jiao Tong University, Shanghai 200444, China

**Keywords:** copolymer sequencing, side-chain liquid crystalline copolymers, charge-induced polyether fragmentation, radical-induced polyether fragmentation, MALDI tandem mass spectrometry

## Abstract

Polyether based side-chain liquid crystalline (SCLC) copolymers with distinct microstructures were prepared using living anionic polymerization techniques. The composition, end groups, purity, and sequence of the resulting copolymers were elucidated by matrix-assisted laser desorption/ionization mass spectrometry (MALDI-MS) and tandem mass spectrometry (MS/MS). MALDI-MS analysis confirmed the presence of (CH_3_)_3_CO– and –H end groups at the initiating (α) and terminating (ω) chain end, respectively, and allowed determination of the molecular weight distribution and comonomer content of the copolymers. The comonomer positions along the polymer chain were identified by MS/MS, from the fragments formed via C–O and C–C bond cleavages in the polyether backbone. Random and block architectures could readily be distinguished by the contiguous fragment series formed in these reactions. Notably, backbone C–C bond scission was promoted by a radical formed via initial C–O bond cleavage in the mesogenic side chain. This result documents the ability of a properly substituted side chain to induce sequence indicative bond cleavages in the polyether backbone.

## 1. Introduction

Side-chain liquid crystalline (SCLC) polymers and copolymers are used in numerous technological applications, such as sensing, transistors, optics, and optical data storage [1,2,3,4]. The mesogenic (i.e., liquid crystalline) side chains endow optical anisotropy, a particularly valuable property in optical devices. Meanwhile, the low fluidity that these molecules possess augments their storage capacity because of increased stability below their glass transition temperature [1,4]. Recent studies have further documented that properly structured SCLC polymers can undergo reversible shape changes in response to external stimuli, like heat, light, or humidity, such controlled deformability is desired for the design and engineering of microactuators and soft robots [5,6,7]. The mentioned utilitarian applications have spurred significant progress in the synthesis of SCLC (co)polymers with low polydispersity (Ɖ), controlled chain length and chain end functionalities, and no or low amounts of byproducts [2,3,4,5,6,7]. Analytical methods that unveil the latter chemical information are essential for optimizing the synthetic routes and conducting meaningful structure-property correlations to design SCLC macromolecules with the desired properties.

The physical properties of SCLC macromolecules can be modulated by introducing two different types of liquid crystalline side chains [8,9]. In such cases, the resulting copolymer may have random, block, alternating, or tapered architecture, depending on polymerization technique and comonomer reactivity. In addition to the structural attributes mentioned above, the sequence of mesogenic comonomers must also be known to understand and improve the physical properties of the synthesized SCLC systems.

In this study, matrix-assisted laser desorption/ionization mass spectrometry (MALDI-MS) [10] and tandem mass spectrometry (MS/MS) [11,12,13,14] are employed to elucidate the compositions, end groups, comonomer content, and sequence of linear SCLC polyether copolymers prepared by anionic ring-opening polymerization of epoxides functionalized with two different liquid crystalline side chains [9,15,16]. Simultaneous as well as sequential addition of the comonomers were employed to generate random versus block architectures, respectively. In earlier studies, the average number of comonomers in such polymers was appraised by ^1^H and ^13^C NMR spectroscopy, their average molecular weights were measured by gel permeation chromatography (GPC) and multi-angle light scattering (MALS), their thermal properties were evaluated by calorimetry, and their self-assembly behavior was examined by X-ray scattering techniques [9,15,16]. Unlike the mentioned analytical methods, mass spectrometry probes individual oligomers, not the average state [14]. It is used here to investigate the SCLC copolymer distributions arising under different experimental conditions. Additionally, MS/MS is applied for the first time to select SCLC oligomers to analyze their comonomer sequences and gain detailed insight about the SCLC microstructures produced by the examined polymerization designs. MS/MS has the sensitivity and selectivity necessary to characterize the comonomer sequence across the entire chain [14,17,18,19,20,21,22,23,24,25,26], which is an important determinant of the ensuing assembly behavior and resulting morphology of the SCLC copolymers.

## 2. Materials and Methods 

### 2.1. Materials

Tetrahydrofuran (THF) and methanol (MeOH) solvents were acquired from Thermo Fisher Scientific (Fair Lawn, NJ, USA), and sodium trifluoroacetate (NaTFA) cationizing agent and trans-2-[3-(4-tert-butylphenyl)-2-methyl-2-propenylidene]malononitrile (DCTB) matrix were acquired from Sigma-Aldrich (St. Louis, MO, USA); all were used as received.

**Scheme 1:**
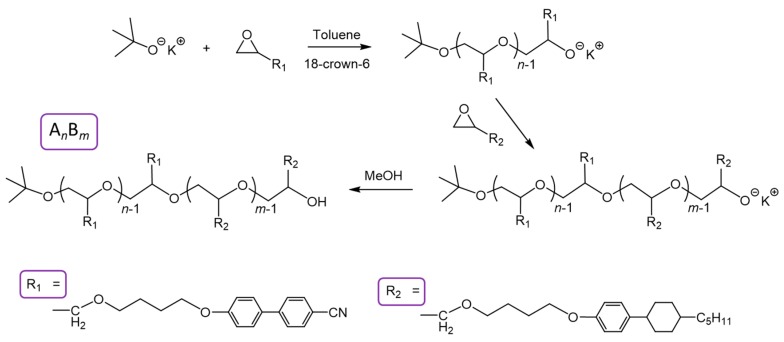
Anionic ring-opening copolymerization of epoxides giving rise to polyethers with liquid crystalline side chains [9,16].

The polymers analyzed were prepared by anionic ring-opening polymerization as has been described in detail in earlier publications [9,16]. Scheme 1 summarizes the synthetic steps used to form block copolymers. Briefly, a given amount of monomer A (determined by the desired length of the A*_n_* block) was polymerized in toluene using 18-crown-6 and potassium tert-butoxide as initiator system. The reaction was maintained for 5 days to consume all A monomer. Monomer B was then transferred into the reaction mixture and the reaction continued for 5 more days. The polymerization was finally terminated by adding MeOH. For a random sequence, equimolar amounts of the comonomers were copolymerized and the reaction was terminated after completion of the polymerization process (i.e., after all monomers had reacted). All products were precipitated into methanol several times and dried before analysis.

### 2.2. NMR and GPC Experiments

GPC with a multi-angle laser light scattering detector (Wyatt Dawn HELEOS II, Wyatt Technology, Santa Barbara, CA, USA) plus a differential refractometer detector (Waters Model 2414, Waters Corp., Milford, MA, USA) was utilized to determine the molecular weight and polydispersity of the copolymers (in THF solvent). ^1^H NMR spectra were recorded on a Varian MERCURY plus-400 (400 MHz) spectrometer (Agilent, Santa Clara, CA, USA) using CDCl_3_ solutions, with chemical shifts reported in ppm relative to the residual deuterated solvent.

### 2.3. MALDI-MS and MS/MS Experiments

MALDI-MS and MS/MS experiments were performed with a Bruker UltraFlex III tandem time-of-flight (ToF/ToF) mass spectrometer (Bruker Daltonics, Billerica, MA, USA) equipped with an Nd:YAG laser (355 nm). Solutions of the polymer and DCTB matrix were prepared in THF at concentrations of 10 and 20 mg mL^−1^, respectively. The NaTFA cationizing agent was dissolved in MeOH at 10 mg mL^−1^. The matrix and cationizing agent solutions were mixed in the ratio 10:1 (*v*/*v*), and approximately 0.5–1.0 μL of the resulting mixture were spotted onto a 384-well ground steel MALDI target plate and allowed to dry under ambient conditions; 0.5–1.0 μL of sample solution was then deposited on top of the dried matrix/salt spot, followed by an additional 0.5–1.0 μL of matrix/salt solution on top of the dried sample spot (sandwich method) [26]. MS/MS experiments were performed using the instrument’s LIFT (laser-induced fragmentation) mode [26,27] with no additional collision gas [28]. Bruker’s FlexAnalysis software was used for data analysis.

### 2.4. Molecular Weight and Comonomer Content Analysis

Average molecular weights (*M*_n_, *M*_w_), polydispersities (Ɖ = *M*_w_/*M*_n_), and comonomer content in the copolymers were calculated using the Polymerix software (Sierra Analytics, Modesto, CA, USA). Raw mass spectral data are input into this program, which deconvolutes homo- and copolymer mixtures to render the chemical formulae of the comonomers and end groups as well as information about the comonomer distributions present across the observed molecular weight range and the corresponding average copolymer compositions.

## 3. Results and Discussion

### 3.1. MALDI-MS Analysis of SCLC Copolymers

The copolymers investigated contain two types of repeat units, viz. A (side chain R_1_) and B (side chain R_2_). They include one block copolymer (sample **1**) with the putative average block sizes A_5_B_13_, suggested by NMR (cf. Figure S1 in Supplementary Material), as well as a random copolymer with unknown composition (sample **2**), prepared using a 1:1 molar mixture of comonomers A and B; all were formed using tert-C_4_H_9_OK for initiation and CH_3_OH for termination to instill tert-C_4_H_9_O– and –H groups at the initiating and terminating chain ends, respectively.

**Figure 1:**
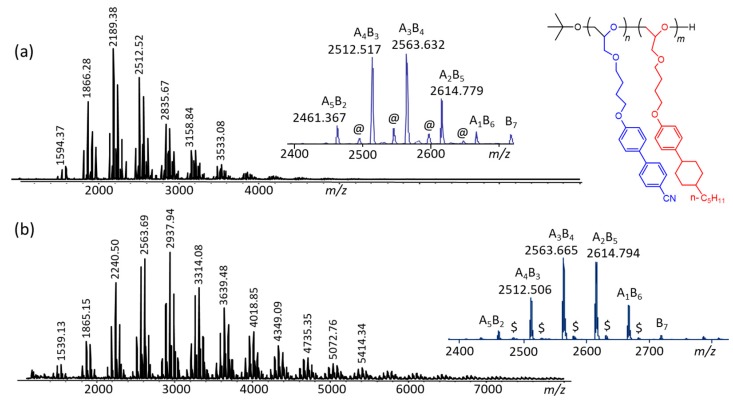
Matrix-assisted laser desorption/ionization mass spectrometry (MALDI-MS) spectra of (**a**) block copolymer **1** and (**b**) random copolymer **2**. All peaks correspond to [M*_p_* + Na]^+^ ions. The insets show the structure of the block copolymer (top right) and expanded views of the *m/z* range 2400–2800, in which heptamers are observed. Calibrated monoisotopic masses and comonomer contents are marked on top of the peaks in the insets (see also Table S1). The sign @ designates methanol adducts with the composition [M*_p_* + Na + CH_3_OH]^+^. The sign $ designates [M*_p_* + K]^+^ ions.

The MALDI-MS spectra of **1** and **2** (cf. Figure 1 and Table S1) confirm the formation of copolymers with the composition tert-C_4_H_9_O-A*_n_*B*_m_*-H, which are detected as sodiated [M*_p_* + Na]^+^ ions (*p* = *n* + *m*). A peak group is observed for each degree of polymerization (*p*), containing oligomers with the same *p* but differing in comonomer content (i.e., in *n* and *m*); the peak groups arising from *p* = 7 (heptamers) are shown in the insets of Figure 1. Within each peak group adjacent A*_n_*B*_m_* oligomers are 51 Da apart, which is the mass difference between repeat units A (323 Da) and B (374 Da); expectedly, the content in the heavier comonomer B increases with increasing mass, as is clearly evident for the heptamers. No other peaks are detected, attesting high product purity.

The MALDI-MS spectrum of the random copolymer **2** includes [M*_p_* + K]^+^ ions with very low relative intensities (cf. inset in Figure 1b), presumably from potassium salt entrapped in the sample during synthesis. Conversely, the block copolymer **1** shows methanol adducts in its MALDI-MS spectrum. Interestingly, this product is not formed from the random copolymer **2**, indicating that a block architecture is necessary to form a stable adduct with methanol, which is most likely attached noncovalently via hydrogen bonding.

The dispersive nature of mass spectrometry allows for monitoring the copolymer composition at the oligomer level (cf. Figure 1). The comonomer content of all A*_n_*B*_m_* polymer chains detected in the MALDI-MS spectra of samples **1** and **2** is listed in Tables S2 and S3, respectively. Average values and important statistical data extracted from these Tables are summarized in Table 1.

Both monomers A and B are glycidyl ether derivatives which have been shown to exhibit very similar anionic polymerization kinetics irrespective of their side chain substituent [29]. This is corroborated by the random copolymer **2**, which was prepared from an equimolar mixture of A and B. Its MALDI-MS data indicate an average copolymer composition with approximately equal numbers of comonomers A and B (cf. Table 1), in agreement with the similar reactivities expected for the glycidyl ether structures A and B.

For the block copolymer, the difference observed in the average block sizes measured by MALDI-MS (Table 1) and NMR (see footnote ^a^ in Table 1) is attributed to potential partial overlap in the NMR signals characteristic for blocks A*_n_* and B*_m_* (cf. Figure S1). There is also a discrepancy between the average molecular weights measured by GPC (cf. Figure S2) and MALDI-MS (Table 1). MALDI-MS may underestimate the molecular weights, although the narrow polydispersity of the copolymers, which is evident from their Poisson-like molecular weight distributions (cf. Figure 1), makes this scenario unlikely [30]. Conversely, GPC may narrow the molecular weight distributions [31,32] and overestimate the average molecular weights [33,34] due to poor detection of the low-mass oligomers [31,32,33,34].

**Figure 2:**
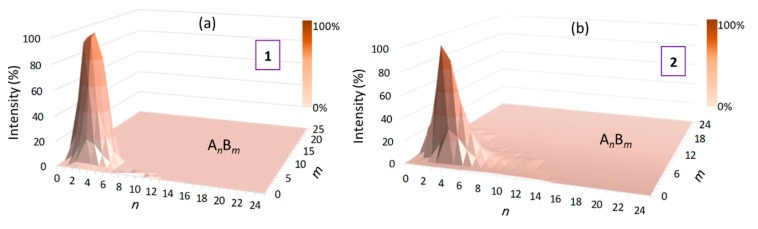
3D intensity maps for the comonomer incorporation in (**a**) block copolymer **1** and (**b**) random copolymer **2**.

The data in Tables S2 and S3 were also used to construct 3D intensity maps that graphically display the A*_n_*B*_m_* comonomer content of the two copolymers (Figure 2). These graphs clearly illustrate the compositional similarity between block copolymer **1** and random copolymer **2**. The graphs and MALDI-MS in general do not reveal the sequence of A and B in the A*_n_*B*_m_* polymer chains. This architectural information can be provided by MS/MS experiments, however, which exploit the intrinsic (unimolecular) fragmentation chemistry of individual cationized oligomers [14]. The sequence indicative dissociations of samples **1** and **2** depend on the structural features of both the polymer backbone as well as the side-chain pendants, as discussed in the following section.

### 3.2. MALDI-MS/MS Fragmentation Pathways of the SCLC Copolymers

**Figure 3:**
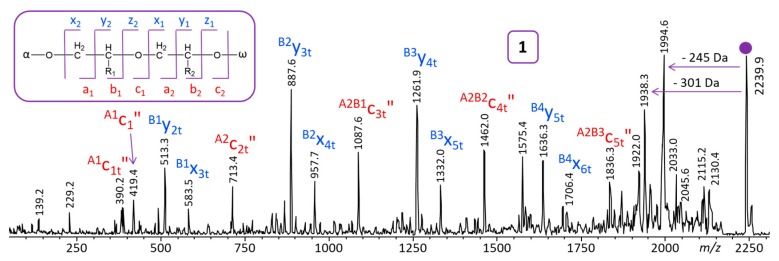
MALDI-MS/MS spectrum of sodiated tert-C_4_H_9_O-A_2_B_4_-H from block copolymer **1** (*m/z* 2239.9). The inset explains the letter code for the fragments from an –O–C–C–O– polyether chain [12].

The sequences of copolymers **1** and **2** can be decoded by the fragments generated upon MS/MS via bond cleavages in the polyether backbone chain. The MALDI-MS/MS spectrum of block copolymer **1** (Figure 3) contains two major contiguous fragment series from such dissociations, one including the initiating tert-butyl chain end (^A*n*B*m*^c*_p_*_t_”) and the other the terminating hydrogen substituent (^A*n*B*m*^y*_p_*_t_). A second, minor fragment series with the terminating hydrogen substituent (viz. ^A*n*B*m*^x*_p_*_t_) is also observed. In the fragment nomenclature used, the letter code (c, x, or y) designates which bond in the polyether chain was broken (cf. inset of Figure 3) and whether the fragment retains the initiating (c) or terminating (y and x) chain end. The subscripted *p* gives the total number of complete or partial comonomer units in the fragment and the superscripted A*_n_*B*_m_* provides the number of complete A and B repeat units in the fragment. The subscripted t tags truncated fragments that are missing part of the side chain or end group, while ” indicates that the dissociation resulted in the formation of a new saturated chain end (otherwise, a double bond was formed at the new chain). For example, fragment ^B2^y_3t_ was produced by C–C bond scission in the polyether chain and contains the segment with the terminating hydrogen substituent; it includes two complete B repeat units plus a truncated unit missing part of the side chain (which could have been A or B); and its new chain end includes a double bond (hence, no ”).

**Scheme 2:**
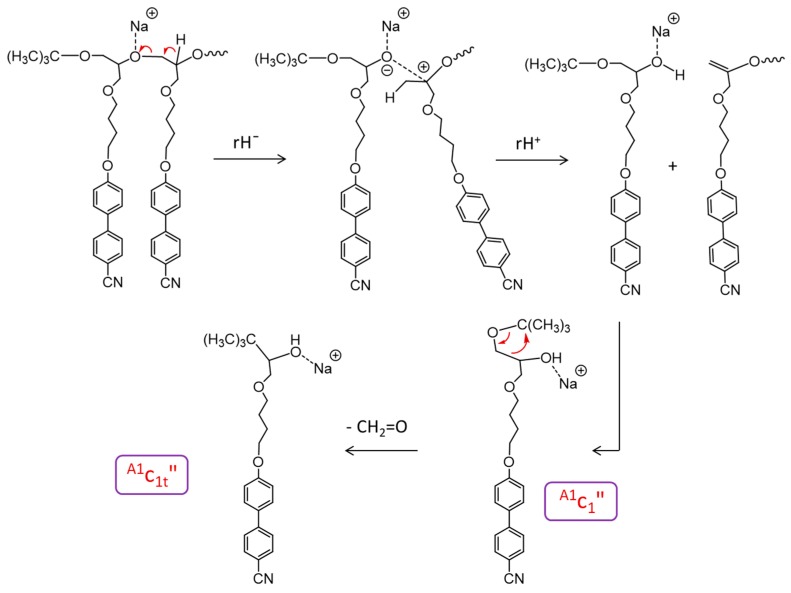
Charge-induced fragmentation pathway leading to the ^A*n*B*m*^c*_p_*_t_” fragments. It is illustrated for the cleavage of the first backbone ether bond, but can occur at any other O–C bond in the polyether chain.

Fragment series ^A*n*B*m*^c*_p_*_t_”, which comprises the tert-C_4_H_9_ substituent from the initiator, is accounted for by random charge-induced dissociations at the backbone ether bonds, catalyzed by the Na^+^ Lewis acid (cf. Scheme 2) [12]. Na^+^ coordination at an ether oxygen can promote heterolytic O–C bond cleavage to form a sodium alkoxylate and a carbocation that interact electrostatically. The incipient carbocation can readily rearrange via 1,2-hydride shift (rH^−^) to a more stable tertiary oxocarbenium cation. Proton abstraction from the latter ion by the alkoxylate anion (rH^+^) gives rise to fragments composed of A*_n_* and B*_m_* comonomer units plus tert-C_4_H_9_O– and –H end groups (i.e., series ^A*n*B*m*^c*_p_*”). Only one such fragment is observed, viz. ^A1^c_1_” at *m/z* 419.4 in Figure 3. All other fragments in this series are truncated by CH_2_O (30 Da), indicating that consecutive loss of CH_2_=O is facile under MALDI-MS/MS conditions [35]. This reaction is rationalized by a rearrangement elimination of formaldehyde from the initiating chain end (cf. Scheme 2 bottom), which leads to the observed truncated ^A*n*B*m*^c*_p_*_t_” fragments, like ^A1^c*_1_*_t_” (*m/z* 390.2) in Figure 3.

**Scheme 3:**
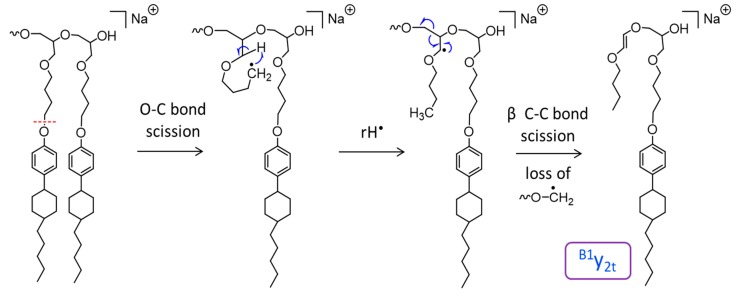
Charge-remote fragmentation pathway leading to the ^A*n*B*m*^y*_p_*_t_ fragments. It is illustrated for a backbone C–C cleavage near the terminating chain end, but can occur similarly at any other C–C bond of the polyether chain.

The most abundant fragment series (^A*n*B*m*^y*_p_*_t_) carries the terminating chain end and is attributed to charge-remote homolytic bond cleavages (cf. Scheme 3) [12]. All ^A*n*B*m*^y*_p_*_t_ fragments are missing one piece of the mesogenic side chain, viz. either NC-C_6_H_4_-C_6_H_4_-O (from repeat unit A) or C_5_H_11_-C_6_H_10_-C_6_H_4_-O (from repeat unit B), strongly suggesting that dissociation is initiated by homolytic O–C bond scission at the aromatic ether of the side chain (cf. Scheme 3). The free radicals created in this step can rearrange to more stable secondary, α-alkoxy functionalized radicals (rH^●^), from where they can induce β C–C bond scission in the polyether chain to form the ultimately observed fragment ions (as depicted for ^B1^y_2t_ in Scheme 3). Note that the resulting fragments contain one truncated mesogenic side chain and their overall composition is A*_n_*B*_m_* + 116 Da + Na^+^.

**Scheme 4:**
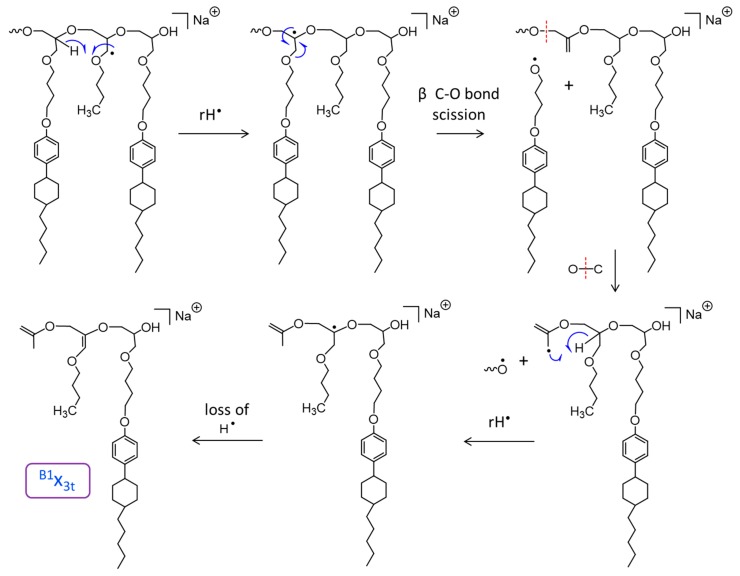
Charge-remote fragmentation pathway leading to the ^A*n*B*m*^x*_p_*_t_ fragments. It is illustrated for a backbone O–C cleavage near the terminating chain end, but can occur similarly at any other similar location of the polyether chain.

The α-alkoxy functionalized radicals (third structure in Scheme 3) may undergo further hydrogen transfer before β bond scission. This alternative mechanism can explain the removal of an additional side chain segment to eventually form the minor fragments ^A*n*B*m*^x*_p_*_t_ via the series of hydrogen rearrangements and homolytic bond cleavages shown in Scheme 4. These fragment ions contain two truncated side chains and have the overall composition A*_n_*B*_m_* + 186 Da + Na^+^.

### 3.3. Sequence Analysis of the SCLC Copolymers by MALDI-MS/MS

For the oligomer A_2_B_4_ from copolymer **1** (Figure 3), the progression ^A1^c_1t_” (*m/z* 390) → ^A2^c_2t_” (*m/z* 713) → ^A2B1^c_3t_” (*m/z* 1088) → ^A2B2^c_4t_” (*m/z* 1462) → ^A2B3^c_5t_” (*m/z* 1836) reveals the sequence AABBB- starting from the initiating chain end. Conversely, the progression ^B1^y_2t_ (*m/z* 513) → ^B2^y_3t_ (*m/z* 888) → ^B3^y_4t_ (*m/z* 1262) → ^B4^y_5t_ (*m/z* 1636) reveals the sequence -BBBB starting from the terminating chain end, which is corroborated by the progression ^B1^x_3t_ (*m/z* 584) → ^B2^x_4t_ (*m/z* 958) → ^B3^x_5t_ (*m/z* 1332) → ^B4^x_6t_ (*m/z* 1706). Combined, these data establish the block sequence AABBBB. Similar analysis of the MS/MS fragmentation pattern of oligomer A_2_B_3_ from copolymer **1** confirms the block sequence AABBB (cf. Figure S3).

**Figure 4:**
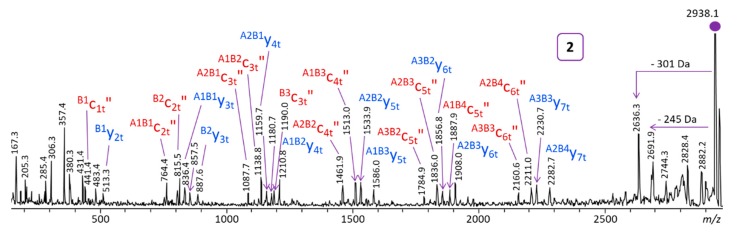
MALDI-MS/MS spectrum of sodiated tert-C_4_H_9_O-A_3_B_5_-H from random copolymer **2** (*m/z* 2938.1).

A common characteristic of the MS/MS spectrum of the block copolymer is the generation of only one fragment of a given degree of polymerization within a series. For example, only one c-type fragment with three comonomer units is observed from A_2_B_4_ (^A2B1^c_3t_” at *m/z* 1088). This allows a straightforward decoding of the sequence based on the mass differences between adjacent fragments of the same series (as discussed above). The fragmentation pattern becomes much more complex for random copolymers, as is evident from the MALDI-MS/MS spectrum of the A_3_B_5_ oligomer from sample **2** (cf. Figure 4). Now, several fragments with the same degree of polymerization but different comonomer content are detected within each fragment series. For example, the MS/MS spectrum of sodiated A_3_B_5_ includes three different c-type fragments with three comonomer units, viz. ^A2B1^c_3t_” (*m/z* 1088), ^A1B2^c_3t_” (*m/z* 1139), and ^B3^c_3t_” (*m/z* 1190) and two different y-type fragments with two complete side chain pendants, viz. ^A1B1^y_3t_ (*m/z* 836) and ^B2^y_3t_ (*m/z* 888). Such behavior diagnoses a random sequence for the A_3_B_5_ oligomer from sample **2**. A completely analogous behavior is observed for oligomer A_4_B_3_ from sample **2** (cf. Figure S4).

## 4. Conclusions

Two different SCLC copolymers were analyzed to gain insight into their compositions and architectures. These important structural features could be conclusively determined by MALDI-MS and MALDI-MS/MS experiments, which require very small sample amounts (<1 mg per copolymer) and short analysis times (a few seconds per spectral acquisition from each copolymer). Sample **2** was confirmed to have a random distribution of the comonomers, while samples **1** was found to be a diblock copolymer.

Sequence analysis requires that the copolymer undergoes bond cleavages in the backbone [14,26,36]. Fortunately, the SCLC polyether copolymers investigated underwent such dissociations under MALDI-MS/MS conditions, thus rendering the sought connectivity information. It is noteworthy, however, that the mesogenic side chains affected the homolytic bond dissociation pathways taking place upon MS/MS. Whereas polyethers with no side chain substituents dissociate preferentially via homolytic C–O bond scissions [12], the SCLC copolyethers underwent homolytic C–C bond scissions in their backbone, which were promoted by radicals created at the mesogenic side chains through facile initial cleavage of (aryl-O)-CH_2_ bonds (cf. Scheme 3). Such a concept, i.e., conjugation of a substituent that can induce homolytic bond cleavages in polymer chains during MS/MS, has been long sought for biopolymer sequencing [37,38]. Our results point out that a long mesogenic substituent, like the side chains in **1** and **2**, may open such fragmentation channels.

The present study showed that MALDI-MS/MS can provide the fragmentation data needed to derive the sequence of singly charged SCLC oligomers with *m/z* ratios up to ~3000. MS/MS analysis of multiply charged ions formed by electrospray ionization (ESI) may extend sequencing capabilities beyond this range [39]. Moreover, multiply charged ions can be energized to dissociate not only thermally (as done here), but also by electron-based activation methods such as electron capture dissociation and electron transfer dissociation [40,41], which often improve sequence coverage [42,43]. Finally, MS/MS can be interfaced with ion mobility separation of either the precursor oligomers and/or their fragments to gain more insight into the microstructures and architectures of SCLC materials [14,39,42]. Future studies will assess the potential of these alternative characterization approaches.

## Figures and Tables

**Table 1:** Average molecular weights and comonomer content of side-chain liquid crystalline (SCLC) copolymers **1** and **2**.

Copolymer	*M*_n_	*M*_w_	Ɖ	Comonomer Composition	
	(Da)	(Da)		Most Abundant	Average
block copolymer **1** ^a^	2874.4	3251.9	1.13	A_3_B_3_	A_3.9_B_4.1_
random copolymer **2** ^b^	3538.7	4184.7	1.18	A_3_B_5_	A_4.8_B_5.1_

^a^ Putative average block size was A_5_B_13_ (by NMR). ^b^ Prepared using an equimolar mixture of comonomers A and B.

## Supplementary Materials

The following are available online at https://www.mdpi.com/2073-4360/11/7/1118/s1. Figure S1: ^1^H NMR spectra of copolymers **1** and **2**. Figure S2: GPC-RI chromatograms of copolymers **1** and **2**. Figure S3: MALDI-MS/MS spectrum of sodiated tert-C_4_H_9_-A_2_B_3_ from block copolymer **1**. Figure S4: MALDI-MS/MS spectrum of sodiated tert-C_4_H_9_-A_4_B_3_ from random copolymer **2**. Table S1: Accurate mass analysis of major oligomers in the MALDI-MS spectra of copolymers **1** and **2**. Table S2: Comonomer content of the tert-C_4_H_9_O-A*_n_*B*_m_*-H oligomers observed in the MALDI-MS spectrum of block copolymer **1** (Figure 1a). The most abundant oligomer has the comonomer composition A_3_B_3_ (100%). Table S3: Comonomer content of the tert-C_4_H_9_O-A*_n_*B*_m_*-H oligomers observed in the MALDI-MS spectrum of random copolymer **2** (Figure 1b). The most abundant oligomer has the comonomer composition A_3_B_5_ (100%).
