# Supplementary material for: Sequencing of Side-Chain Liquid Crystalline Copolymers by Matrix-Assisted Laser Desorption/Ionization Tandem Mass Spectrometry
Source: Polymers (Basel). 2019 Jul 1;11(7):1118. doi: 10.3390/polym11071118 (PMC6680698; doi:10.3390/polym11071118)
Supplement: Supplementary file 1 [file polymers-11-01118-s001.pdf]

polymers

Supplementary Material for:

Sequencing of side-chain liquid crystalline copolymers by matrix-assisted laser desorption/ionization tandem mass spectrometry

Savannah R. Snyder [1], Wei Wei [2], Huiming Xiong [2], and Chrys Wesdemiotis [1,*]

1 Department of Chemistry, The University of Akron, Akron, OH 44325, United States; srs119@zips.uakron.edu (S.R.S.), wesdemotis@uakron.edu (C.W.)
2 Department of Polymer Science, School of Chemistry and Chemical Engineering, Shanghai Jiao Tong University, Shanghai 200444, P.R. China; wei1330538@sjtu.edu.cn (W.W.), hmxiong@sjtu.edu.cn (H.X.)
* Correspondence: wesdemiotis@uakron.edu (C.W.), Tel.: +1-330-972-7699

Table of contents:

Figure S1. ^{1}H NMR spectra of the SCLC copolymers investigated.

Figure S2. GPC data of the SCLC copolymers investigated.

Figure S3. MALDI-MS/MS spectrum of sodiated tert-C_4H_9O-A_2B_3-H from block copolymer **1**.

Figure S4. MALDI-MS/MS spectrum of sodiated tert-C_4H_9O-A_4B_3-H from random copolymer **2**.

Table S1. Accurate mass analysis data.

Tables S2-S3. Comonomer composition of the SCLC copolymers investigated.

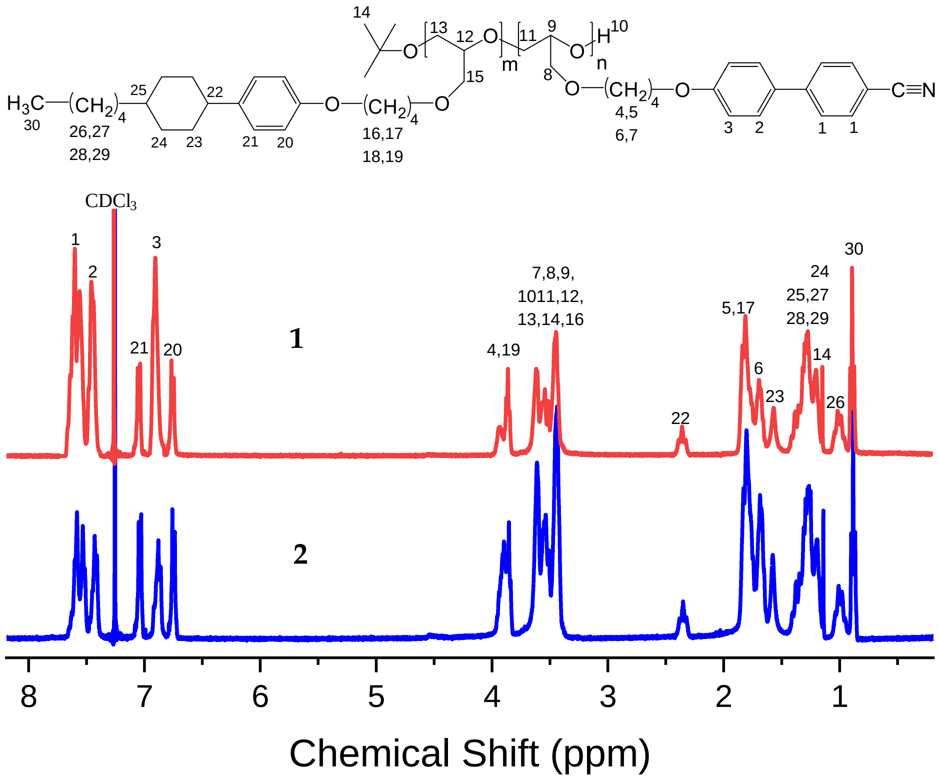

Figure S1. ^{1}H NMR spectra of block copolymer (sample **1**, red trace) and random copolymer (sample **2**, blue trace) in $CDCl_3$.

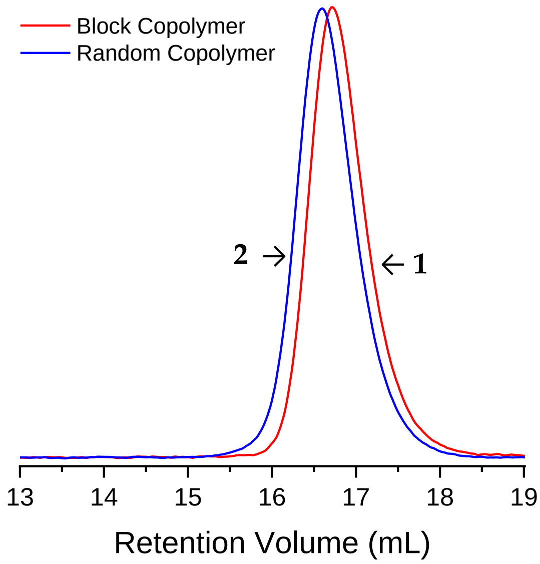

Figure S2. GPC-RI chromatograms of block copolymer (sample **1**, M_w = 6.3 kDa, Đ = 1.05) and random copolymer (sample **2**, M_w = 6.8 kDa, Đ = 1.08).

polymers

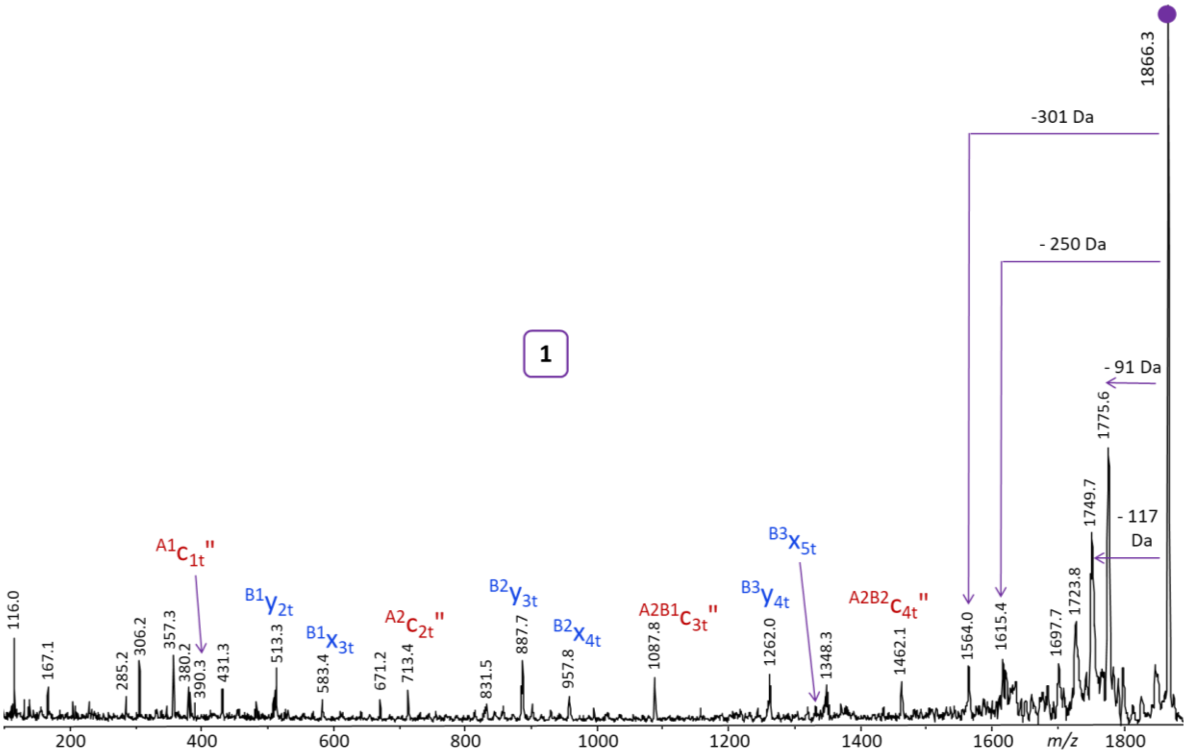

Figure S3. MALDI-MS/MS spectrum of sodiated tert-C_4H_9O-A_2B_3-H from block copolymer **1** (*m/z* 1866.3).

The progression ${}^{A1}c_{1t}''$ (*m/z* 390) → ${}^{A2}c_{2t}''$ (*m/z* 713) → ${}^{A2B1}c_{3t}''$ (*m/z* 1088) → ${}^{A2B2}c_{4t}''$ (*m/z* 1462) reveals the sequence AABB- starting from the initiating chain end. Conversely, the progression ${}^{B1}y_{2t}$ (*m/z* 513) → ${}^{B2}y_{3t}$ (*m/z* 888) → ${}^{B3}y_{4t}$ (*m/z* 1262) reveals the sequence -BBB starting from the terminating chain end, which is corroborated by the progression ${}^{B1}x_{3t}$ (*m/z* 583) → ${}^{B2}x_{4t}$ (*m/z* 958) → ${}^{B3}x_{5t}$ (*m/z* 1332). Combined, these data establish the block sequence AABBB.

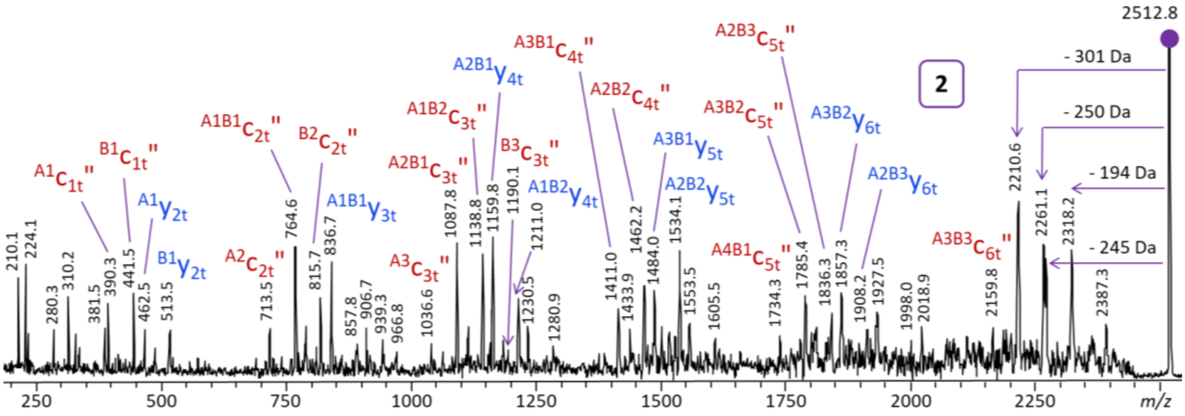

Figure S4. MALDI-MS/MS spectrum of sodiated tert-C_4H_9O-A_4B_3-H from random copolymer **2** (*m*/*z* 2512.8).

As with oligomer A_3B_5 (cf. Figure 4), several fragments with the same degree of polymerization but different comonomer content are detected within each fragment series. For example, this MS/MS spectrum includes four different c-type fragments with 3 comonomer units, viz. ${}^{A3}c_{3t}''$ (*m*/*z* 1037), ${}^{A2B1}c_{3t}''$ (*m*/*z* 1088), ${}^{A1B2}c_{3t}''$ (*m*/*z* 1139), and ${}^{B3}c_{3t}''$ (*m*/*z* 1190); and two different y-type fragments with 3 complete side chain pendants, viz. ${}^{A2B1}y_{4t}$ (*m*/*z* 1160) and ${}^{A1B2}y_{4t}$ (*m*/*z* 1211). Such behavior diagnoses a random sequence for the A_4B_3 oligomer from sample **2**.

polymers

Table S1. Accurate mass analysis of major oligomers in the MALDI-MS spectra of copolymers **1** and **2**.

		$[M_p + Na]^+$ ($p = n + m$)			
		**block copolymer 1**		**random copolymer 2**	
**A_nB_m Oligomer** [a]	***m/z* calcd.** [c]	***m/z* exptl.**	**ppm** [d]	***m/z* exptl.**	**ppm** [d]
A_3B_3 + CH_3OH [b]	2221.392	2221.391	0.5		
A_2B_4	2240.496	2240.496	0.0	2240.497	0.4
A_4B_3	2512.518	2512.517	0.4	2512.506	4.8
A_2B_5	2614.778	2614.779	0.4	2614.794	6.1
A_4B_4	2886.800	2886.798	0.7	2886.782	6.2
A_3B_5	2937.930	2937.931	0.3	2937.942	4.1

[a] All oligomers have the connectivity tert-C_4H_9O-A_nB_m-H.

[b] Noncovalent methanol adduct (observed only for block copolymer **1**, cf. Figure 1a and text).

[c] For the corresponding sodiated species.

[d] Agreement between experimental and calculated *m/z* values.

polymers

Table S2. Comonomer content of the tert-C_4H_9-A_nB_m-H oligomers observed in the MALDI-MS spectrum of block copolymer **1** (Figure 1a). The most abundant oligomer has the comonomer composition A_3B_3 (100%).

A*n*	B*m*																					
	m = 0	1	2	3	4	5	6	7	8	9	10	11	12	13	14	15	16	17	18	19	20	21
n = 0		0.04	0.26	0.91	6.43	18.96	12.65	4.27	1.94	2.75	4.76	3.32	1.66	0.82	0.47	0.34	0.3	0.28	0.31	0.17	0.13	0.11
1	0.04	0.3	0.48	5.02	28.55	27.98	8.58	2.61	1.32	1.89	3.04	2.29	1.2	0.7	0.39	0.32	0.26	0.28	0.26	0.14	0.11	
2	0.18	0.41	1.71	47.62	71.95	34.84	8.66	2.35	1.19	1.42	2.04	1.48	0.93	0.52	0.37	0.26	0.21	0.23	0.2	0.12		
3	0.33	0.45	15.21	100	72.89	26.05	6.43	1.99	0.95	1.04	1.23	1.07	0.66	0.43	0.3	0.22	0.21	0.19	0.18			
4	0.45	0.73	29.16	87.33	49.51	16.49	4.32	1.51	0.74	0.75	0.84	0.7	0.56	0.37	0.26	0.23	0.18	0.17				
5	0.57	0.96	23.44	52.33	28.41	9.81	3.09	1.19	0.64	0.53	0.59	0.55	0.42	0.3	0.24	0.18	0.15					
6	0.59	1.22	12.43	27.76	15.71	6.02	2.18	0.98	0.55	0.44	0.4	0.4	0.32	0.28	0.2	0.17						
7	1.08	1.29	6.68	13.62	8.83	3.92	1.67	0.79	0.46	0.35	0.34	0.3	0.29	0.23	0.19	0.14						
8	1.17	1.32	3.64	6.89	5.01	2.56	1.35	0.67	0.38	0.3	0.26	0.27	0.24	0.2	0.16							
9	1.53	1.17	2.18	3.89	3.03	1.75	0.98	0.54	0.35	0.25	0.23	0.22	0.2	0.17								
10	1.53	1.07	1.39	2.15	1.96	1.22	0.7	0.41	0.3	0.21	0.2	0.18	0.17									
11	1.33	1.04	0.96	1.33	1.2	0.86	0.55	0.37	0.25	0.21	0.17	0.17										
12	1.27	0.73	0.83	0.86	0.81	0.79	0.44	0.31	0.24	0.18	0.16											
13	1.18	0.72	0.55	0.64	0.56	0.46	0.35	0.29	0.21	0.17												
14	1.1	0.6	0.46	0.42	0.42	0.34	0.31	0.24	0.18													
15	0.83	0.53	0.38	0.36	0.3	0.3	0.25	0.2	0.16													
16	0.69	0.44	0.33	0.28	0.25	0.24	0.2	0.17														
17	0.54	0.35	0.27	0.22	0.21	0.19	0.18															
18	0.46	0.32	0.23	0.21	0.17	0.17																
19	0.36	0.26	0.22	0.17	0.15																	
20	0.29	0.25	0.19	0.16																		
21	0.27	0.21	0.16																			
22	0.12	0.09	0.06																			
23	0.09	0.08																				
24	0.08																					

polymers

Table S3. Comonomer content of the tert-C_4H_9-A_nB_m-H oligomers observed in the MALDI-MS spectrum of random copolymer **2** (Figure 1b). The most abundant oligomer has the comonomer composition A_3B_5 (100%).

A*n*	B*m*																							
	m = 0	1	2	3	4	5	6	7	8	9	10	11	12	13	14	15	16	17	18	19	20	21	22	23
n = 0				1.23	1.59	10.97	10.01	5.22	2.71	1.62	1.5	1.81	1.45	8.11	1.08	1.72	0.9	1.06	1.89	0.78	0.74	0.79	0.39	0.56
1			1.32	1.39	38.56	56.7	40.54	21.25	9.08	3.19	1.5	1.13	3.21	1.36	0.83	1.16	1.68	0.74	0.59	0.08	0.1	0.26	0.06	0.12
2		1.46	1.13	39.93	93.23	96.17	71.41	41.52	17.06	1.2	1.08	1.82	3.18	0.83	2.37	1.26	0.64	0.6	0.55	0.18	0.19	0.25	0.28	
3	1.12	1.07	14.68	63.14	97.15	100	81	47.78	1.17	1.16	1.68	6.02	0.87	2.26	0.85	0.65	0.71	0.55	0.54	0.17	0.33	0.22		
4	1.06	1.89	17.35	46.7	71.74	80.28	59.68	2.27	1.18	1.59	3.24	0.94	1.31	0.83	0.74	4.23	3.03	0.55	0.49	0.08	0.08			
5	0.88	2.33	9.7	25.23	42.35	46.87	6.8	1.29	1.55	1.61	0.94	1.89	0.83	5.17	3.82	2.32	1.93	0.56	0.43	0.06				
6	0.82	1.74	4.61	11.51	19.84	8.44	1.22	1.63	1.84	1.77	1.82	0.8	5.08	0.87	0.69	0.82	0.71	0.44	0.55					
7	1.21	1.48	2.58	4.86	4.81	1.16	1.39	1.63	6.19	1.23	1.21	1.16	0.84	0.68	0.55	0.75	1.24	0.43						
8	2.66	2.27	2.18	2.27	1.33	1.46	1.23	4.04	0.96	2.53	0.75	0.73	0.63	0.53	0.51	0.63	0.41	0.89						
9	5.05	3.64	2.37	1.81	3.85	1.09	2.1	1.23	1.68	0.73	0.94	1.83	1.51	0.5	0.65	1.3	0.44							
10	6.35	4.38	2.11	14.59	1.06	1.75	4.24	1.17	0.72	3.22	2.41	2.71	1.78	0.48	0.65	0.39								
11	4.9	1.99	32.88	1.08	1.43	10.44	0.91	1.78	3.22	1.06	0.87	2.25	0.46	0.51	0.5									
12	1.87	41.52	1.13	1.28	10.67	0.89	7.86	1.11	1.2	0.93	0.71	1.23	0.46	0.57										
13	26.21	1.55	1.29	2.62	0.9	7.95	1.26	0.78	0.64	0.66	0.6	0.72	0.4											
14	4.58	1.18	1.35	0.86	1.82	0.93	0.74	0.7	0.56	0.5	0.62	0.4												
15	1.13	1.22	0.86	0.92	0.77	0.95	0.84	1.14	0.54	0.53	1.42	0.47												
16	1.04	1.13	0.84	1.32	0.83	0.92	0.74	1.94	0.45	0.58	0.38													
17	1.52	0.77	2.76	1.1	1.04	0.91	0.86	0.75	0.54	0.41														
18	0.79	1.57	1.2	0.73	0.67	0.85	1.69	0.46	0.67															
19	1.53	0.8	0.69	0.59	0.6	0.53	0.46	0.38																
20	0.74	0.65	0.57	0.6	0.53	0.69	0.4																	
21	1.87	1.64	0.56	0.69	0.43	1.17																		
22	0.54	0.29	0.34	0.1	0.09	0.19																		
23	0.61	0.74	1.37	0.54	0.39																			
24	0.6	1.13	0.22	0.61																				
25	0.57	0.62	0.24																					
26	0.85	0.39																						
27	0.8																							
